# Supplementary material for: Are carbon emissions trading and green financial instruments synergistic? -Comprehensive quantitative research based on content analysis
Source: PLoS One. 2024 Mar 7;19(3):e0298601. doi: 10.1371/journal.pone.0298601 (PMC10919601; doi:10.1371/journal.pone.0298601)
Supplement: S1 Data — (ZIP) [file pone.0298601.s001.zip › Data/地级市Z_F工作报告.zip!/║╙─╧/╫ñ┬φ╡Ω╩╨/└┤╫╘╣1⁄2╓┌║┼í╛┬φ┐╦╩2╛▌═°í┐_2016.docx]

各位代表：

现在，我代表市人民政府，向大会报告工作，请予审议，并请各位政协委员和其他列席人士提出意见。

一、“十二五”重大成就及“十三五”主要目标

回顾过去的五年，我们走过的历程很不平凡。面对复杂严峻形势和一系列重大风险挑战，在省委、省政府和市委正确领导下，全市上下全面落实党的十八大和十 八届三中、四中、五中全会精神，深入贯彻习近平总书记系列重要讲话精神，紧紧围绕富民强市加快崛起目标，开拓创新、真抓实干，胜利完成了“十二五”规划主 要目标任务，经济社会发展取得了令人鼓舞的重大成就!

综合实力稳步提升。去年全市生产总值达到★1807.1亿元(加★包括新蔡县，其他不包括，下同)，年均增长9.7%，人均生产总值由14112元提高到26024元;规模以上工业增加值575亿元，年均增长15.3%;一般公共预算收入★96亿元，年均增长21.4%;社会消费品零售总额699亿元，年均增长14.8%;粮食生产在高基点上实现新跨越，总产达★151.8亿斤，实现 “十二连增”。

转型升级步伐加快。三次产业结构从★27.5:41.8:30.7调整为22.3:40.1:37.6，二、三产业比重提高 5.2个百分点，其中三产 比重提高6.9个百分点。科学载体功能不断完善，带动能力持续提升，产业集聚区规模以上工业增加值、主营业务收入分别占全市的79%和82%;服 务业“两区”建设步伐加快，一批千万元以上商业项目建成投用，集聚效应更加凸显。全市城镇化率达39.2%，比“十一五”末提高9.4个百分点，中心城区 建成区面积由53平方公里扩大到85平方公里，人口由48万增加到近70万。创新能力持续增强，建立院士工作站3个、博士后科技工作站2个，省级以上工程 技术研发中心、企业技术中心、工程实验室64个，高新技术企业26家。生态环境明显改善，万元生产总值能耗比“十一五”末下降15%，主要污染物排放总量 全面完成省定目标。

基础支撑能力不断增强。固定资产投资累计完成近5000亿元，是“十一五”时期的2.5倍。交通、水利、能源、信息等一批重大项目建成投用，全市综合 交通线网里程超过1.84万公里，其中高速公路通车里程437公里，实现所有县20分钟上高速。实施了宿鸭湖、薄山、铜山湖3座大型水库和160多座中小 型水库除险加固，完成了小洪河等19条中小河道治理。吴桂桥煤矿、华润风电等重大项目建成投运，电力总装机达到230万千瓦，实现220千伏变电站九县全 覆盖。信息网络不断完善，全市乡镇以上区域实现4G网络全覆盖。

改革开放成效显著。行政体制改革稳步推进，简政放权持续加快，行政审批事项大幅缩减。商事制度改革全面实施，新登记企业数量快速增长。政府机构改革和 事业单位改革稳步推进，交通运输行政执法和食品药品监管机构改革全面完成。医药卫生体制改革深入推进，实现了城镇居民大病保险市级统筹和新农合县级统筹。 农村综合改革和财税、水利、科技、教育、文化等领域改革取得积极进展。开放招商成效显著，累计到位各类资金1855.7亿元，为“十一五”时期的4倍，其 中实际利用外资15.1亿美元、利用省外资金898.5亿元。

人民生活明显改善。城镇居民人均可支配收入22608元，农民人均可支配收入9174元，年均分别增长11.1%和13.4%。五年新增城镇就业39 万人，新增农村劳动力转移就业44万人。企业退休人员养老金和城乡低保标准连年提高，城镇居民医疗保险参保率、新农合参合率分别达97%和98%，覆盖城 乡的社会保障体系初步形成。义务教育均衡发展取得新成就，九年义务教育巩固率达95%。卫生事业加快发展，新增医疗卫生机构床位8600多张。公共文化服 务体系不断完善，推出了一批群众喜闻乐见的精品力作。新建改建农村公路3296公里，解决48.9万农户“低电压”问题，改造农村危房9.3万 户，69.7万贫困人口稳定脱贫，人民群众福祉显著提高。

各位代表!回顾五年的奋斗历程，我们迎难而上、负重前行，实现了经济社会健康快速发展，为全面建成小康社会奠定了坚实基础，我市的发展已站在了新的历 史起点上。取得这样的成就来之不易，这是全面落实党中央、国务院和省委、省政府决策部署的结果，是市委坚强领导的结果，是市人大、市政协大力支持的结果， 是全市人民团结奋斗的结果。在此，我代表市人民政府，向广大工人、农民、知识分子、干部，向驻驻部队指战员、武警官兵、公安民警及社会各界人士，表示崇高 的敬意和衷心的感谢!

这五年，我们在现代化建设实践中积累了宝贵经验，主要是：坚持贯彻中央和省委决策部署与立足驻马店实际相结合，坚定正确政治方向;坚持发展第一要务，凝心聚力加快发展步伐;坚持“四化”同步推进，统筹经济社会协调发展;坚持深化改革开放，激发发展动力和活力;坚持以人为本，着力保障和改善民生;坚持推进伟大工程，切实加强和改善党的领导。这些经验是全市人民智慧的结晶，也是我们开创未来的制胜法宝，必须倍加珍惜、发扬光大!

五年的成就和经验，凝聚着全市干部群众的聪明才智和辛勤汗水，极大增强了全市人民的自信心和自豪感，必将激励我们在新的征程中奋勇前进!

各位代表!“十三五”是全面建成小康社会的决胜阶段，也是驻马店实现富民强市加快崛起的关键时期，必须保持定力、攻坚克难，以滚石上山的精神和勇气，奋力开拓加快发展新局面。

根据市委建议，市政府编制了《驻马店市国民经济和社会发展第十三个五年规划纲要(草案)》，已印发会议，提请审议。

“十三五”时期经济社会发展的指导思想是：高举中国特色社会主义伟大旗帜，全面贯彻党的十八大和十八届三中、四中、五中全会精神，以马克思列宁主义、 毛泽东思想、邓小平理论、“三个代表”重要思想、科学发展观为指导，深入贯彻习近平总书记系列重要讲话精神，按照“五位一体”总体布局和“四个全面”战略 布局，坚持发展第一要务，牢固树立创新、协调、绿色、开放、共享发展理念，以提高经济发展质量和效益为中心，着力创新体制机制、增强发展动力，着力推进四 化同步、统筹协调发展，着力优化经济结构、转变发展方式，着力深化改革开放、厚植发展优势，着力保障改善民生、共享改革发展成果，全力打造“四个驻马 店”，确保如期全面建成小康社会，为实现富民强市加快崛起奠定更加坚实基础。

主要预期目标是：在提高发展平衡性、包容性、可持续性基础上，生产总值和城乡居民人均收入比2010年翻一番以上，主要经济指标年均增速高于全省平均水平，力争主要人均指标达到或接近全省平均水平;转 型升级和创新驱动取得新进展，改革开放开创新局面，人民生活得到新提高，全民素质和社会文明达到新水平，生态环境质量实现新改善，治理体系和治理能力现代 化迈出新步伐，到2020年全面建成小康社会，基本形成现代化框架格局，综合竞争优势大幅提升，成为综合实力较强、人民生活富足、生态文明良好的区域性豫 南中心城市，富强文明平安美丽驻马店展现出更加美好前景!

实现上述目标，必须坚持创新发展，培育发展新动力;坚持协调发展，构筑发展新格局;坚持绿色发展，建设美丽新家园;坚持开放发展，拓展发展新空间;坚持共享发展，创造幸福新生活。

各位代表!宏伟的目标召唤着我们，人民的期待激励着我们。我们坚信，经过五年的不懈努力，我们一定能够圆满完成全面建成小康社会这一伟大历史任务!二、2015年工作回顾

2015 年是“十二五”的收官之年。一年来，我们按照市委的决策部署，紧紧围绕“推发展、促改革、保稳定、抓党建、惠民生”五项重点工作，着力做强载 体、做大产业、做实项目、做好招商、做优环境，经济社会发展保持良好态势，较好完成了市三届人大六次会议确定的目标任务。全市生产总值增长8.9%;规模以上工业增加值增长9.2%;固定资产投资增长19.4%，居全省第二;社会消费品零售总额增长13.3%，居全省第一;一般公共预算收入增长 12.1%;城镇居民人均可支配收入、农村居民人均可支配收入分别增长8.7%和10%，主要经济指标增速均高于全省平均水平。重点抓了以下工作：

(一) 努力保持工业稳定增长。坚持把工业发展作为稳增长的重中之重，认真落实上级各项政策措施，深入开展工业“稳增长、调结构、防风险、保发展”活 动，实施了稳增长保态势重大工作专项、出台了推进企业创新发展实施意见、设立企业应急周转专项资金，健全企业服务协调机制，并通过召开经济运行分析会、现 场办公会等形式，加强企业服务，强化产销、银企、用工对接，协调解决企业发展中的问题，促进了工业经济的稳定增长。全市规模以上工业企业主营业务收入增长 12.8%，实现利润增长10.5%，其中主营业务收入超120亿元企业1家，超10亿元企业21家。电动汽车、电子信息、新材料等战略新兴产业加快发 展，新增国家高新技术企业13家，战略新兴产业企业发展到64家。深入开展产业集聚区晋档升级活动，大力发展主导产业和产业集群，全市产业集聚区完成固定 资产投资970.3亿元、实现主营业务收入2102亿元，分别占全市的73%和82%。

(二)着力加大项目建设力度。深入开展项目建设推进年活动，继续实行四大班子领导分包重点项目制度，认真落实模拟审批、联审联批、全程代理等工作机 制，采取观摩点评、现场办公、早餐会等形式协调解决问题，深入开展中心城区重点项目征地拆迁集中行动，累计拆迁218万平方米，为项目落地创造了条件。全 年分四批集中开工153个亿元以上重点项目，总投资740亿元，年度投资214亿元。鹏辉锂电池、鼎复康药业等一批重点项目竣工投产，德威电缆、顺达化工 醋酸制乙醇等一批重大项目开工建设，金龙沃诚二期、后弈制药等一批续建项目加快推进;京港澳高速驻马店段改扩建工程顺利竣工，107国道升级改造及干线公路、农村公路项目加快推进;病险水库除险加固、小农水重点县、中小河流治理等水利项目进展顺利。全市22个省重点项目完成投资112亿元，占年度计划的 123%;264个市重点项目完成投资770亿元，占年度计划的119%，其中194个亿元以上工业项目完成投资501亿元，占年度计划的126%，均超额完成目标任务。

(三)强力推进开放招商。坚持把开放招商作为“一举求多效”的重要举措，继续实行重大招商项目领导分包和月通报制度，强化合同履约率、资金到位率、项 目开工率督察，进一步提高招商引资实效。深入开展大招商活动，强化产业招商、定点招商、落地招商、以商招商，先后派出300余支招商小分队，举办各类招商 活动15次，累计签约亿元以上项目248个，合同投资1628.5亿元，其中10亿元以上项目53个，合同投资981.4亿元。成功举办第十八届中国农加 工洽谈会，现场签约合同项目76个，总投资696.5亿元，项目质量和投资规模较往年均有新提高。市公共保税物流中心“两仓”封关运营，全年到位省外资金 222.4亿元，增长9%;完成进出口4.5亿美元，增长11.3%，增幅分别居全省第二位和第三位。

(四)加快推进服务业发展。坚持把服务业发展作为优化产业结构的重点，进一步完善措施，强化政策支持，努力弥补发展短板。加快推进“两区”建设，完成 投资近60亿元，建成区面积4.66平方公里。认真落实国家、省促进房地产业发展政策措施，出台住房公积金使用和购房、契税补贴优惠政策，全市房地产投资 额、销售收入分别增长15%和8.9%。加快旅游业发展，嵖岈山景区成功创建国家5A级景区，全市旅游综合收入134.7亿元，增长26.5%。加快金融 业发展，金融机构各项存款余额2237.6亿元，增长12.9%;各项贷款余额1115.5亿元，增长18.7%，余额存贷比50%，新增存贷比68%;招行引资、企业上市取得突破，兴业银行驻马店支行开业运营，蓝天燃气等5家企业实现“新三板”挂牌。电子商务产业加快发展，各类电商企业2000多家，交易额156.8亿元，增长124%;阿里巴巴、中国网库、京东商城等50余家电商企业成功入驻，阿里巴巴·驻马店产业带上线企业突破600家，线上线下交易额突破10亿元。现代物流、健康养老、“互联网+”等产业加快发展，服务业发展活力明显增强。

(五)统筹推进新型城镇化。强化“一基本、两牵动、三保障”， 不断创新投融资机制，加强基础设施和公共服务设施建设，城镇承载能力进一步提高，城镇化率达39.2%，提高1.7个百分点。年初确定的中心城区100多个重点城建项目顺利实施，累计完成投资46.3亿元;抓 住政策机遇，启动市区驿城大道贯通、薄山水库引水及市科技馆、青少年宫、会展中心、第四污水处理厂等重点项目前期工作。切实加强城市规划和土地管理，加大 “双违”整治力度，进一步规范土地供应、规划审批和项目建设秩序。全面启动“六城联创”工作，开展环境卫生“百日综合整治”和“百日重点治理”活动，实施 38条“断头路”打通、210条背街小巷改造、环卫设施完善提升工程，城市面貌发生较大变化。加快城乡一体化示范区发展，理顺管理体制，谋划实施一批重点 项目。各县围绕县城和重点镇建设，实施一批旧城改造、片区开发、城镇基础设施和公共服务项目，城镇功能逐步完善，城镇面貌发生很大变化。

(六) 持续推进现代农业发展。切实加强高标准粮田和农业基础设施建设，新建高标准粮田105.2万亩，全年粮食总产151.8亿斤，增长5.1%，实 现“十二连增”。稳步推进适度规模化经营，土地流转面积占家庭承包面积的31%。加快农业产业化发展，农业产业化集群达到55个，其中省级15个;市 级以上重点龙头企业351家，其中国家级2家、省级46家。积极培育新型农业经营主体，农民合作社达到13550家、家庭农场5021家，数量居全省前 列。改善农村人居环境工作扎实推进，创建省级达标村563个、示范村261个。现代畜牧业持续发展，新建标准化规模养殖场99个，生猪饲养量居全省第一， 肉牛、家禽饲养量居全省第二。夏南牛产业发展取得突破，存栏在50万头以上，以河南恒都食品为龙头的肉牛产业化集群加快发展，恒都公司成为首批澳洲进口活 牛主要承接企业，年屠宰加工15万头专用生产线正在加紧建设。

(七)稳步推进重点领域改革。坚持问题导向，以解决影响经济健康发展突出障碍、影响民生保障和社会公平突出问题、影响社会稳定突出隐患为重点，积极跟 进落实中央、省改革举措，稳步推进各项改革。着力转变政府职能，全面取消非行政许可审批，再次减少行政审批事项78项，精简29%，市政府工作部门全部建 立权责清单。全面推行“三证合一、一照一码”商事登记制度。深化行政体制改革，完成市、县两级政府机构和事业单位分类改革，交通运输行政执法体制和食品药 品监管体制改革全面完成，产业集聚区管理套合改革顺利推进。大力推进投融资体制改革，建立企业应急贷款周转金、土地出让金周转金，推行融资担保、政府购买 服务、股权债券融资、争取国家专项建设基金等投融资方式，融资渠道全面拓宽。深入推进财税体制改革，新预算法得到全面贯彻。加快金融改革发展，完成驻马店 银行参与中原银行组建工作，西平农商行挂牌营业，市农商行、遂平农商行组建工作有序推进，遂平县“两权抵押”贷款试点获得批准。继续深化国有企业改革，9 家企业进入改制程序，其中3家完成改制任务。县级公立医院改革全面推开，药品加成全面取消。农村集体建设用地使用权、农村宅基地确权颁证超额完成年度任 务。18个试点村土地承包经营权确权颁证全面完成。户籍制度改革全面推进，不动产统一登记制度改革、机关事业单位养老保险制度改革全面启动，党政机关公务 用车制度改革顺利实施，教育、文化等领域的改革取得新进展。

(八)切实保障和改善民生。进一步加大民生投入力度，“十项重点民生工程”和中心城区六件惠民实事顺利推进，全市民生支出269亿元，占一般公共预算 支出的80%。全力推进精准扶贫，对121个贫困村实施整村推进扶贫开发，10.8万农村贫困人口实现稳定脱贫。加强就业和社会保障工作，完成各类劳动力 技能培训16.5万人，新增农村劳动力转移就业6.7万人，新增城镇就业7.8万人;完 善失业、医疗、工伤、生育保险市级统筹，提高低保、城乡居民基础养老、企业退休人员养老和五保供养标准，启动实施城镇居民大病保险和机关事业单位养老保 险，新农合参合率、新农保参保率均达98%。新开工保障性住房3.4万套，基本建成2万套，分配入住1.8万套，完成省定任务。全面发展社会事业，新建、 改扩建幼儿园513所，学前三年毛入园率达82%;认真实施农村义务教育全面改薄和城镇扩容规划，改造学校148所;持续推进全民技能振兴工程和职教二期工程，中职学校全日制在校生学费全免，市职教园区完成投资6.4亿元，驻马店技师学院挂牌成立;卫 生事业发展改革三年九大攻坚行动计划圆满收官，人均基本公共卫生服务经费补助标准达到40元。市体育中心、气象科普馆等项目顺利推进，成功举办市二运会暨 首届全民健身大会。深入实施蓝天、碧水、乡村清洁工程，加快推进安全饮水等项目建设，完成农村危房改造2.7万户。残疾人事业持续发展，成功创建全国残疾 预防示范市。妇女儿童、慈善、红十字等事业健康发展。广泛开展舞台艺术送农民、文化艺术进社区活动，文化惠民演出实现城乡全覆盖。重拳打击和分类处置非法 集资，扎实开展安全生产排查整治活动，全力保障食品药品安全，集中化解信访突出问题，严厉打击违法犯罪活动，保持了社会大局和谐稳定，人民群众的安全感、 满意度持续上升。

一年来，我们自觉接受人大监督、政协民主监督和社会监督，认真办理人大代表建议和政协提案，支持民主党派、工商联和无党派人士参政议政。切实加强民主 法制工作，深入推进依法行政和服务型行政执法，认真落实行政执法责任制，法治政府建设取得新进展。深入开展“三严三实”专题教育和“三查三保”活动，持续 推进反腐倡廉建设，严格落实中央八项规定和省市若干意见，健全权力运行制约和监督体系，强化行政监察和审计监督，专项治理懒政怠政为官不为，着力解决不严 不实和不作为乱作为等损害群众利益问题，风清气正、干事创业氛围更加浓厚。加强国防教育、国防后备力量和人民防空建设，驻驻解放军、武警部队和民兵预备役 人员为地方发展作出新贡献，双拥工作深入推进，军民融合深度发展，军政军民团结更加巩固。民族宗教、外事侨务、统计、气象、史志、对口援疆等工作取得新成 绩。

各位代表!在肯定成绩的同时，我们也清醒认识到，当前我市经济社会发展中还存在一些突出问题：一是经济总量小，产业结构不合理。生产总值总量在全省排 名靠后，经济结构与全国、全省有较大差距，一产比重大，工业仍是经济发展的关键性短板。二是城镇化水平低，以城带乡能力较弱。城镇化率远低于全国、全省平 均水平，中心城区和县城服务功能不完善，产业支撑能力不强，对农民转市民的吸纳保障能力较弱，对农村的辐射带动力不强。三是农业现代化水平较低，农村发展 仍很滞后。粮经饲比例不够合理，农业产业化、规模化水平较低，农村基础设施和公共服务落后，人居环境脏乱差问题突出，农村贫困人口脱贫任务艰巨。四是现代 金融体系落后，金融服务能力不足。现代金融体系不健全，引进股份制银行数量少，村镇银行、金融租赁、基金、信托等发展滞后，利用资本市场直接融资能力较 弱，融资难、融资贵问题仍很突出。五是公共服务投入不足，保障水平较低。农村优秀教师流失严重，教育薄弱;城市教育发展滞后于城市扩张，大班额现象严重;基层医疗服务能力弱，分级诊疗水平不高;健 康养老产业发展滞后，与群众期待有较大差距。六是发展环境不优，稳定任务繁重。政府部门作风转变还不到位，一些干部不负责、不作为、不担当、懒政怠政，甚 至以权谋私、贪污腐败现象依然严重，项目落地难、群众办事难问题仍很突出。诚信法治的社会环境还不健全，违法建设、干扰施工现象时有发生。同时，我市安全 生产、生态环境、社会稳定等压力大、风险大。对此，我们一定高度重视，勇于担当，认真解决，努力让全市人民满意!三、2016年重点工作

今年是实施“十三五”规划的开局之年。综合判断我市面临的外部环境，可以说是机遇与挑战同在、希望与困难并存，总体来看机遇大于挑战。一方面，世界经济仍处于深度调整疲弱复苏状态，不确定性不稳定性较大;我 国经济发展进入新常态，增速放缓、动力转换、供给侧结构不合理、经济下行压力大，形势严峻复杂。受宏观形势的影响，我市部分行业困难加剧，一些企业经营困 难，房地产库存较大、销售疲软，财政收入增长放缓，民间投资能力和意愿下降，资源环境约束加大，各类潜在风险累积叠加。另一方面，党的十八届五中全会、中 央经济工作会议、中央城市工作会议和中央农村工作会议，确立了引领新常态的“五大发展理念”，明确了“五大政策”和今年的“五大经济任务”。围绕“十三五 规划”、国家脱贫攻坚规划以及覆盖我市全境的大别山振兴发展规划的实施和供给侧结构性改革，国家将出台一系列稳增长、促发展的政策措施，必将为我们争取更 多的资金项目支持带来重大机遇;长三角、珠三角腾笼换鸟加快，京津冀协同发展深化，进一步增强了产业转移的动力;我省实施的“三大国家战略”和主动融入 “一带一路战略”，加之我市的交通区位和资源要素优势，进一步增强了承接产业转移的竞争力;新一轮科技革命和产业变革蓬勃兴起，大众创业、万众创新与“互联网+”、智能机器人、“中国制造2025” 融合互动发展，为我们加快发展电子商务、现代物流、智能制造等产业带来了新机遇;“四 化”同步发展蕴藏的巨大需求潜力不断释放，尤其是传统农区去产能包袱小、发展潜力大，为我们加快发展、实现“弯道超车”提供了机遇。面对经济新常态下复杂 严峻的新形势，我们一定要清醒认识，保持定力，既要有打持久战的耐心和韧劲，又要有善抓机遇、顺势而为的紧迫感和责任感，坚定信心、下定决心，振奋精神、 脚踏实地，努力推动全市经济社会发展再上新台阶，确保“十三五”开好局、起好步。

今年经济社会发展的主要预期目标是：生产总值增长在 8.5%左右，一般公共预算收入增长8.5%，全社会固定资产投资增长16%，社会消费品零售总额 增长12%，进出口总值增长8%，实际利用市外资金增长20%，城镇新增就业5万人以上，城镇化率提高1.5个百分点，城镇居民人均可支配收入增长8%， 农村居民人均可支配收入增长9%，居民消费价格涨幅控制在2%左右，节能减排、生态环保等约束性指标完成省定目标，发展的质量和效益明显提高。

生产总值预期增长在8.5%左右，充分考虑了我市完成全面建成小康社会目标的要求，也客观估计了新常态下发展的可能性。特别是经过近年来的发展，我们已经有了一定的基础，具备加快发展的条件，只要我们同心同德，真抓实干，实现这一目标是完全可能的。

一般公共预算收入预期增长8.5%，与市委经济工作会议确定的9%下调0.5个百分点，主要基于以下考虑：现在已是2月下旬，中央和省今年的工作部署已陆续到位，形势政策更加明朗，财政收入形势比年初预期更加严峻;营改增改革全面推开，对房建两税收入影响较大;国 家推进供给侧结构性改革要实行大幅度减税降费政策，降成本减轻企业负担还要进一步清理取消减少收费，这些政策措施的实施将大幅减少财政收入。今年，全省财 政收入预期增长7%，低于经济增长1个百分点，周边市也下调了财政收入增幅。综合权衡，将一般公共预算收入预期增速确定为8.5%是事实求是的，也是可行 的。

做好今年的政府工作，要全面落实市委各项决策部署，牢固树立创新、协调、绿色、开放、共享发展理念，主动适应经济发展新常态，更加注重稳增长、促改 革、调结构、强基础、惠民生、防风险，着力优化结构做大做强产业，着力开放招商承接产业转移，着力统筹城乡推进新型城镇化，着力扩大投资加强基础设施建 设，着力深化改革创新驱动发展，着力改善民生维护社会稳定，加快推进“四个驻马店”建设和“四化”同步发展，确保实现“十三五”良好开局，为全面建成小康 社会打下坚实基础。

(一)着力优化结构，做大做强产业。抓住国家推进供给侧结构性改革的机遇，坚持需求导向，加快结构调整，促进产业升级，持续提升产业竞争力。

实施“工业强市”战略，加快推进工业经济发展。坚持以产业集聚区建设为载体，大力推进工业强市战略，促进工业经济加快发展。大力改造提升传统产业。加 强传统产业和骨干企业技术改造和链条延伸，强化上下游企业合作，全面落实“中国制造2025河南行动”和“互联网+工业”行动计划，大力推进信息技术在产 品研发、节能减排、创新发展、产业服务等方面的应用，不断提升传统行业竞争力，力争食品、轻纺、装备制造、能源煤化工、建材、生物医药等六大支柱产业主营 业务收入分别达到750亿元、650亿元、610亿元、430亿元、270亿元、80亿元。加快发展高成长性和战略性新兴产业。围绕汽车及零部件制造、电 子信息、装备制造业、食品等高成长性产业，继续强化政策扶持，重点支持重大技术攻关、骨干企业智能化改造和产业基地建设，促进产品由产业链低端向高端提 升，形成全产业链竞争优势。巩固海川电子玻璃、银泰新能源汽车、中云创光电等行业领先优势，抓好德威电缆、华顺阳光光伏发电、金龙沃诚电动汽车等项目建 设，引领和推进新能源汽车、新型建材、光伏光热、环保设备等战略新兴产业发展。打造培育优势产业集群。深入实施“百千亿级”产业集群培育工程，依托我市优 势产业和骨干企业，科学制定产业发展规划，着力引进产业链上下游和关联配套企业，着力打造食品千亿级产业集群和轻纺、能源化工、装备制造、生物医药等百亿 级产业集群。

转变发展方式，加快推进现代农业发展。坚持集约、高效、绿色、可持续发展，进一步优化农业结构，转变发展方式，加快推进现代农业发展。提高粮食产能和 质量。全面落实藏粮于地、藏粮于技战略，实行最严格耕地保护制度，持续推进高标准粮田“百千万”建设工程，实施耕地质量保护与提升行动，新建高标准粮田 111.5万亩。抓好农田水利建设，新增和恢复有效灌溉面积98万亩，新增节水灌溉面积50万亩，新增旱保田面积64万亩。

加强优良品种的培育引进推广，开展粮食绿色高产高效创建，巩固提高粮食产能，不断提升粮食品质，确保粮食产量稳定在140亿斤以上。调整优化农业结 构。推进农业供给侧结构性改革，加快种植结构调整，因地制宜发展特色农业、高效农业、绿色农业、循环农业和品牌农业，增加市场紧缺农产品生产，提高农产品 供给质量和效益。大力发展规模养殖，巩固畜牧业发展优势，推动粮经饲统筹、农林牧渔协调。积极发展都市生态农业，支持有条件的地方发展乡村特色经济园区， 拓展农业功能，提高农业综合效益。加强农资和农产品市场建设与管理，严厉打击和处置假冒伪劣产品，维护农民群众利益。促进农业产业化规模化发展。坚持种养 加一体、一二三产业融合发展，推动农业产业化经营、标准化生产、品牌化营销。加快培育农业产业化集群，持续推进夏南牛、黑猪、食用菌、花生、芝麻等特色产 业集群发展，新增省级农业产业集群3个、市级20个。加快推进正阳国家级农业科技示范园区建设，发挥好示范引领作用。有序推进土地适度规模经营，积极培育 农民合作社、家庭农场、农业产业化企业等新型农业经营主体和新型职业农民，探索农民合作社产品订单、大型农用生产设施及土地承包经营权、林权等财产抵 (质)押贷款办法，提高农业发展融资能力，力争新增省级农业产业化龙头企业5家、市级30家，新型农业经营主体数量增长10%。提高农业科技装备水平。加 强农业科技创新和推广应用，强化现代农业产业和技术体系建设。不断提升农业机械装备水平，积极推进智慧气象和农业遥感技术的应用。积极探索物联网、“互联 网+”在农业领域的应用，大力发展“互联网+现代农业”。

依托人口区位优势，加快推进现代服务业发展。把发展现代服务业作为产业转型升级的重点，依托我市区位、人口、市场和资源优势，推动现代服务业加快发 展。加快生产性服务业发展。大力发展互联网经济和电子商务产业，依托市、县电子商务产业园，支持阿里巴巴·驻马店产业带、中国网库驻马店电商谷、楚楚街电 商总部等企业在我市加快发展，积极引进和承接百度、腾讯等知名电商来我市搭建平台、拓展业务;鼓 励我市商贸、工业企业线上交易，扶持发展一批“电商村”、“淘宝村”，打造我市电子商务产业发展新高地。加快现代物流业发展，着力推进粮食、医药、化工、 冷链、快递等优势物流业发展，重点抓好恒兴物流电子商务产业园、驻马店国际公路物流港等项目建设，编制完成豫南物流园区规划，加快推进铁路物流产业园前期 工作。深入推进供销社综合体制改革，大力发展连锁经营企业、配送中心、标准农家店、村级综合服务社，全面提升流通网络现代化水平。积极推动文化创意、规划 设计、勘测评估、会计律师等生产性服务业发展。提升生活性服务业水平。大力发展旅游产业，加快旅游精品线路和旅游景区规划建设，加强嵖岈山5A旅游景区规 划建设和管理，加大宣传推介力度，巩固创建成果;保护利用好嵖岈山、金顶山、老乐山、铜山和薄山湖、宿鸭湖、铜山湖、板桥水库“四山四湖”生态旅游资源;加快推进杨靖宇纪念馆改扩建和竹沟红色小镇建设，开发利用好红色旅游资源;加 快推进皇家驿站建设，加大天中文化、驿站文化、盘古文化、嫘祖文化、女娲文化、重阳文化、冶铁铸剑文化、车舆文化、南海禅寺佛教文化以及西游、梁祝等传说 文化宣传力度，挖掘利用好文化旅游资源，全市旅游业综合收入突破160亿元。加快现代金融业发展，继续推进金融体系建设，新引进2家股份制银行或城市商业 银行设立分支机构;加快确山县、汝南县村镇银行组建步伐;完成驻马店农商行组建工作，推动遂平县、汝南县农商行获批，泌阳县、上蔡县、平舆县农联社达到筹建标准;加强上市企业培育，实现6家企业在“新三板”挂牌;创新金融产品，争取信贷规模，确保全市各项贷款余额存贷比在50%以上，新增存贷比在60%以上;积极引进保险、信托、金融租赁、基金等非银行金融机构;积 极探索信用保险、贷款保证保险服务小微企业融资新途径。创新发展文化产业，完成文化产业发展规划编制工作，制定文化产业发展意见，扎实推进重点文化企业培 育工程，积极推动文化与科技、金融、旅游、互联网融合发展。大力发展健康养老产业，抓好市城乡一体化示范区健康养老产业园、老乐山银发壹族养生养老园、泌 阳康复养老服务中心等项目规划建设。主动适应电商时代需要，不断提升专业市场、超市、餐饮、住宿等传统服务业发展水平。促进房地产业健康发展。把鼓励住房 消费、加快消化库存作为一项重要任务，支持缴存职工用公积金贷款改善住房条件，落实好商品房购房补贴和契税补贴政策，加大公积金归集扩面力度，将有稳定劳 动关系的常住人口纳入覆盖范围，促进新市民在城镇购房，鼓励农民进城购房。建立购租并举住房制度，加快推进保障性住房货币化安置，畅通商品房供给和保障性 住房通道;培育发展住房租赁市场，公租房新增需求主要通过发放货币化补贴、政府购买或长期租赁商品房解决。坚持分类调控、精准调控，优化土地供应结构，防控商业地产风险。

强化企业服务，增强发展活力。重点围绕企业经营运行和创新发展搞好服务，提升服务企业的能力和水平。认真落实扶持政策。积极开展降低实体经济企业成本 行动，全面落实国家和省降低企业税费负担、社会保险费、电力价格等政策。鼓励企业加强内部管理挖潜，实现降成本、增效益。因企施策搞好服务。坚持因企施 策、分类指导，对有竞争力、有销路企业，帮助解决好融资、用工等生产要素保障，促进开足马力生产;对生产经营困难企业，综合运用延伸链条、技术改造、淘汰落后产能等手段，帮助企业摆脱困境;对重点企业实行动态管理，继续落实领导分包制度，加强经济运行监测，做好煤电油气运协调和要素保障，发挥好骨干支撑作用。创新政府支持方式。继续开展银企、产销、用工、产学研对接，着力解决企业发展中的困难和问题;积极引导市内企业强化市场联合，互为市场解决销路问题，不断提升企业抗风险能力;积极运用基金参与、购买服务、股权投资等方式，支持龙头企业发展和项目建设。加大对创新型企业和战略性新兴产业发展在资金、政策等方面的支持力度。

(二)统筹城乡发展，积极推进新型城镇化。认真落实中央城市工作会议精神，综合施策、加大力度，着力推动城镇化提速提质发展。

有序推进农业转移人口市民化。推进新型城镇化要坚持以人为核心，解决好进城农民落户、居住、就业和各项权益保障问题。加快推进户籍制度改革。建立城乡 统一的户口登记制度，全面放开县城和建制镇落户限制，进一步放开中心城区落户条件，努力提高户籍人口城镇化率。落实好居住证制度。对非户籍常住人口实行居 住证制度，建立以居住证为载体、与居住年限等条件相挂钩的基本公共服务提供机制。维护好进城落户农民权益。完善进城落户农业人口原有权益处置机制，维护好 进城农民土地承包权、宅基地使用权、集体收益分配权，积极探索建立农村宅基地有偿退出机制;完善进城落户农业转移人口就业保障、住房保障、随迁子女接受教育、社会保障等政策制度，实现公共服务均等化。加快推进产业集聚区、服务业“两区”建设，大力发展城区经济和县域经济，增强产业支撑能力。

提升城镇规划建设管理水平。遵循城市发展规律，统筹推进中心城区、县城和重点镇建设，全面提升城镇规划建设管理水平。坚持高起点规划。依据城市总规要 求，按照“一中心五组团”和中心城区、县城、乡镇所在地三级体系布局，坚持以人为本、“多规合一”原则，不断创新规划理念，统筹做好生产、生活、生态等功 能布局，加强城市设计和控规、修规编制与执行，完善市政设施、公共服务、防灾避险、生态休闲等专项规划。重视城乡一体化示范区、开发区、产业集聚区、服务 业“两区”、专业园区、旅游景区等功能片区规划的编制，提高规划的完整性和可执行性。坚持高标准建设。加快中心城区建设，实施好城市道路、生态水系、公园 绿地、环卫设施、公共管线及照明五大类263个基础设施项目。全面完成38条“断头路”续建和新打通25条“断头路”任务，完成市区置地大道、重阳大道下 穿京广铁路项目，加快推进老火车站片区综合改造工程。加快推进薄山水库引水工程和第三水厂建设、板桥水库引水管道更新和取水口改造、第一二水厂供水管网改 造工程，确保中心城区供水安全。加强中心城区道路、供排水、强弱电、热力燃气、垃圾污水处理、环卫、停车场等市政基础设施建设。加快练江河、小清河、开源 公园、十三香公园建设进度，实施好中心城区生态引水和水系连通工程，完善城市生态休闲功能，建设“海绵”城市。进一步加强旧城区和棚户区改造，加快推进市 城乡一体化示范区、中心城区七大片区开发建设，积极推进学校、医院、养老等公共服务设施建设。大力推进县城扩容提质工程，加强基础设施建设，强化产业支 撑，不断提升县城的承载吸纳能力。以改善农村人居环境和实施重点镇建设示范工程为重点，加强乡镇所在地基础设施和公共服务设施建设，更好地为农业和农村发 展服务。坚持高水平管理。以创建国家文明城市、卫生城市和森林城市为重点，深入开展“六城联创”活动，加强基础设施和公共服务设施建设，进一步加大交通秩 序、环境卫生、市容市貌和背街小巷、集贸市场、早夜市摊点、旱厕改造等专项整治力度。不断完善中心城区“两级政府、三级管理、四级网络”管理长效机制，积 极推进城市综合执法体制改革，加快推进智慧城市建设，促进城市管理规范化、精细化、网格化、智慧化。积极实施提升县级城市管理水平三年行动计划，深入开展 各项创建活动，打造优美人居环境，不断增强城镇的宜业宜居性。

科学推进新农村建设。按照”五规合一”要求，统筹和优化村庄、产业、用地、基础设施和公共服务设施、生态保护等布局，科学编制新农村建设规划。加大农 村水、电、路、气、房等基础设施投入力度，持续开展农村人居环境综合整治，实施农村生活垃圾治理五年专项行动，加强农村生活垃圾收集转运和生活污水收集处 理等环卫设施建设，探索建立村庄保洁长效机制，积极推进美丽宜居乡村示范创建，确保今年20%的行政村达到人居环境达标村标准、10%的行政村达到示范村 标准。

(三)实施投资拉动战略，强力推进项目建设。坚持把项目建设作为扩大投资、加快发展的重要抓手，进一步加大项目建设力度，确保全市固定资产投资突破1500亿元。

积极争取项目。认真学习研究上级政策，围绕交通体系、粮食水利、新型城镇化、脱贫攻坚、生态环保以及自主创新、结构调整、社会事业等重点领域和产业， 积极谋划包装一批重大项目。做好项目的论证和可研，加大向上级部门的沟通对接力度，争取得到更多项目和资金支持，为我市长远发展打牢坚实基础。

科学安排项目建设计划。加强项目库建设。对筛选确定的项目，建立项目库，实行动态管理，合理安排项目实施计划，做到实施一批、筹备一批、筛选论证一 批，保持项目建设的持续性。今年，全市初步确定重点项目234个，总投资2119亿元，年度投资581.7亿元。其中，工业项目156个，总投资 942.9亿元，年度计划投资339.6亿元。高度重视工业重点项目建设，对华顺阳光年产1200万支全玻璃真空太阳集热管、天中食品产业园改造、今麦郎 食品工业园、君乐宝等计划新开工项目，加大协调力度，落实开工条件，确保尽早开工建设;对德威电缆、银泰汽车二期、金龙沃诚二期、后羿制药二期、恒都食品产业园二期等在建项目，加快建设进度，确保项目快推进、早建成;对天方生物年产4万吨木糖醇、顺达化工年产20万吨醋酸制乙醇等计划竣工项目，倒排工期、加快进度，确保项目按期竣工投产;加快天方药业、华中正大搬迁改造进度，力争尽早实现整体搬迁。同时，围绕企业技术改造、转型升级、扩能增效，再新上一批亿元以上工业项目。

高效组织好项目实施。加快项目规划、环评、土地等审批事项办理，积极推进重点项目征地拆迁，为项目落地打好基础。认真落实重点项目领导分包和早餐会、 议事会等制度，加强指导协调，解决好项目实施中的困难和问题。认真落实重点项目月通报、季督导、半年检查和年终考评制度，落实奖惩措施，加快项目进度。加 大环境整治力度，严厉打击破坏发展环境行为，特别是对那些执法犯法、吃拿卡要的典型案件以及封门堵路、干扰施工、恶意阻扰项目建设的组织者、操纵者和黑恶 势力，坚决依法打击。

(四)实施开放带动战略，积极承接产业转移。坚持把开放招商作为拉动投资、扩大内需的关键性举措，大力实施开放带动战略，力争全年实际到位市外资金550亿元。

突出招商引资重点。围绕长三角、珠三角和京津冀等产业转移比较集中的重点区域开展招商，积极承接产业转移;围 绕优势产业、主导产业和产业集群开展招商，通过引龙头和延链补链，做大做强食品、煤化工、装备制造、生物医药、电动汽车、电子信息、电缆电器、节能环保、 新材料、新能源、纺织服装等主导产业和产业集群。拓宽招商引资领域，围绕电子商务、现代物流、现代金融等新业态、新产业开展招商。

改进招商引资方式。认真分析产业转移形势，研判产业转移动向，科学编制《产业招商图谱》，更加突出领导招商、精准招商、以商招商、落地招商，充分发挥 各地行业协会、商会的作用，促进招商活动常态化，特别对一些重大项目，主要领导亲自挂帅，组织专业团队进行落地招商，力争在招大引强上取得更多突破。

利用好活动载体。积极参加国家和省组织的招商活动，主动融入“一带一路”、郑州航空港综合经济实验区等国家战略，努力拓展我市对外开放的广度和深度。 利用好中国农加工洽谈会、保税物流中心、产业集聚区、服务业“两区”等平台，定期举办招商推介会，开展有针对性的产业招商活动，积极承接产业转移，集中引 进一批全产业链、集群式重大项目，不断提升招商水平。同时，积极做好产品销售、劳务输出、进出口以及招才引智等工作，努力发展更高层次的开放型经济。

(五)厚植发展优势，蓄积发展后劲。利用好我市交通区位优势、劳动力和土地要素优势、农业资源优势，通过加强基础设施建设、打造发展平台、强化职业教育和劳动力培训等措施，厚植发展优势，提升经济发展的支撑力和竞争力。

加强基础设施建设。积极争取南驻阜铁路纳入国家规划，开工建设周驻南、息邢高速，完成京港澳高速驻马店站、驻马店南站改造和驻马店北站建设任务。全面 完成G107驻马店段、G240驻平交界至驻南交界段、G230正阳中心庙至驻信交界段、S218正阳县城至驻信交界段等道路升级改造任务，开工建设 G328平舆至驿城区段。以实施城市组团连通工程为重点，加快推进驿城大道南北贯通、铜山大道南延工程;启动开源大道和重阳大道东至宿鸭湖西至七蚁路项目、雪松大道西至七蚁路改造升级项目;开 工建设老乐山东山门至G107、金顶山北山门至G328工程。积极实施农村公路“乡村畅通”工程三年行动计划，建设农村公路1125公里，力争全年完成交 通项目投资42亿元以上。启动中心城区、平舆、遂平嵖岈山、泌阳通用机场规划建设前期工作。积极推进宿鸭湖水库清淤扩容、洪汝河水系连通等项目前期工作， 力争年内开工建设。大力推进泌阳、确山、遂平等新能源发电项目建设，加快华润2×100万千瓦机组、1000千伏驻马店特高压输变电工程前期工作。抓好环 境生态、信息通讯设施等项目建设。

提升载体平台优势。按照五规合一、四集一转、产城互动要求，全面完成产业集聚区管理套合改革，持续推进基础设施和配套公共服务建设，培育壮大主导产 业，全市产业集聚区完成固定资产投资1100亿元，完成主营业务收入2200亿元，1~2个产业集聚区达到二星级。加快推进城乡一体化示范区建设，完成综 合交通体系等10个专项规划、高铁西片区控制性详规编制及核心区城市设计，做好休闲度假旅游、都市生态农业等功能片区规划建设;启 动会展中心、高铁广场地下停车场、商务中心区核心区地下连通工程、高铁西广场区道路等基础设施项目，确保全年完成投资40亿元以上。加快市商务中心区、驿 城区皇家驿站及各县服务业“两区”建设，确保全市完成“两区”投资80亿元以上，在建千万元以上项目60个以上。做好驻马店海关、商检口岸筹建工作，积极 争取市公共保税中心获批。积极推进“中国名优农产品交易网”跨境交易平台建设，着力打造对外开放新平台。发挥好“中国农加工洽谈会”的平台作用，继续做好 今年会议筹办工作，力求取得更大成效。

强化要素保障能力。持续推进全民技能振兴工程和职教攻坚工程，加快人力资源大市向人力资源强市转变。依托黄淮学院、驻马店职业技术学院、驻马店技师学 院和一批中等职业学校，加强校企合作和产学研结合，以企业需求和产业发展需要为导向，增强培养培训的针对性、实用性。实施好阳光雨露计划，整合各部门培训 资源，加强农业转移劳动力的技能培训。实施回归工程，吸引外出务工创业人员和驻马店籍大学生返乡就业兴业。落实好引进人才的优惠政策，支持企业加强院士工 作站、博士后流动站等平台建设，加大招才引智力度，引进一批金融、现代物流、电子商务等紧缺人才，吸引集聚一批科技领军人才和高水平创新管理人才，完成引 进培养高技能人才4500人，各类职业技能培训16万人次。珍惜土地资源优势，强化土地管理，利用好增减挂钩、人地挂钩、占补平衡等政策，盘活存量、优化 增量、集约节约，增强发展用地保障能力。依托农业优势，进一步优化结构、提高质量，扩大农产品资源优势，大力推进一二三产业融合发展，努力将资源优势转化 为产业优势。同时，加快金融、信息产业发展，弥补要素短板。

(六)深化改革创新，增强发展动力。围绕“一跟进、两聚焦、三重点”，不断深化重点领域和关键环节改革创新，着力构建有利于加快发展的体制机制。

深化重点领域改革。继续深化行政审批制度改革,全面建立权责清单制度，加快行政审批标准化建设，积极推广“互联网+政务服务”。完成政府机构改革，加 快事业单位改革，深化商事制度改革，进一步放宽市场准入，加强事中事后监管。深化国有企业改革，实施国有企业功能分类，已进入改制程序企业抓紧向前推进， 其他企业逐一研究制定改革方案、尽快组织实施;全面清理限制非公有制经济发展的不合理规定，保护好民营企业各项权益，促进非公有制经济健康发展。深化财税体制改革，推进“营改增”全面实施和消费税、资源税费改革;继续做好预算管理、政府性债务管理、财政存量资金盘活、政府购买公共服务等工作。积极推进投融资体制改革，组建市投融资集团，提高市县区联动融资能力;大 力推行政府与社会资本合作PPP模式和政府购买服务，创新政府建设公共设施、提供公共服务的方式。积极推进城市综合执法体制改革。进一步理顺产业集聚区、 城乡一体化示范区等经济功能区的体制机制。完成农村土地承包经营权确权登记颁证工作，基本完成农村集体建设用地使用权和宅基地确权登记发证工作。统筹抓好 不动产登记、国有林场、供销社、社会保障、教育、文化、医疗等改革。

促进万众创新。加大资金扶持力度，发挥黄淮学院大学生创新创业园、国家级众创空间、驻马店科技孵化器、中科院河南育成中心驻马店分中心的作用，积极推 进各类众创平台建设，力争全年新建10家市级以上研发平台，其中省级研发平台2家以上，组织实施省级科技重大专项2个，确保规模以上高新技术产业增加值增 长15%，科技进步对经济社会发展的贡献率较上年提高0.5个百分点。

支持大众创业。按照“非禁即入、便民高效、规范统一、宽进严管”原则，凡是国家法律法规未禁止进入的行业和领域，都允许和鼓励各类资本参与投资;凡法律法规未明确禁止的服务行业和经营项目，全部向社会资本开放，鼓励各方创业投资。深入实施大学生创业引领计划，完善和落实支持农民工返乡创业的政策措施,抓好市级创业孵化园建设，全面落实小微企业税收优惠政策，拓宽创业融资渠道，积极推进创业带动就业。

(七)大力实施精准扶贫，打好脱贫攻坚战。认真贯彻中央和省委、市委扶贫开发会议精神，落实精准扶贫、精准脱贫基本方略，围绕我市脱贫攻坚目标，全力以赴、精准发力，确保完成今年全市173个贫困村、10.12万农村贫困人口稳定脱贫。

落实“六个精准”。以农村贫困人口全部脱贫为目标，坚持产业为基、就业为本，大力实施精准脱贫攻坚工程，抓好精准识别、建档立卡工作，建立精准脱贫数 字管理系统，推进脱贫工作信息化、动态化管理。集中力量攻坚，切实做到扶持对象精准、项目安排精准、资金使用精准、措施到户精准、因村派人精准、脱贫成效 精准，确保完成年度脱贫目标任务。

坚持“五条途径”。坚持向城镇转移就业脱贫，力争完成培训转移贫困人口2.1万人，产业集聚区转移就业 1万人，实现自主创业脱贫1万人;坚持发展特色优势产业脱贫，把扶贫开发与推进农业现代化结合起来，实施“一乡一业”、“一村一品”扶贫计划，统筹使用涉农资金，积极推进土地流转，大力发展特色产业，鼓励发展规模适度的家庭农场、农民专业合作社，实现产业脱贫6万人;坚持易地搬迁脱贫，对确山、泌阳、西平、遂平、驿城区24个乡镇、194个贫困村，坚持群众自愿、积极稳妥原则，因地制宜选择搬迁安置方式，合理确定住房建设标准，完善搬迁后继扶持政策，确保搬迁对象有业可就、稳定脱贫，做到搬得出、稳得住、能发展、可致富;坚持社会保障兜底脱贫，把丧失劳动能力的贫困人口纳入低保体系，实行政策性兜底扶贫;坚持扶危济困脱贫，对因突发灾祸可能返贫致贫群众，及时给予临时救急和社会救济，帮助他们渡过难关。积极对接《大别山革命老区振兴发展规划》，科学制定我市具体实施意见，抢抓政策机遇，超前谋划项目，加强沟通衔接，努力向上争取更多资金和项目支持。

采取“五项举措”。实施知识技能扶贫，继续深入实施全民技能振兴工程和职教攻坚工程，提高劳动者素质，提高贫困群众的就业致富能力;实施合作带动扶贫，发挥产业化龙头企业、农业合作社、新型农民等新型经营主体的引领作用，解决小生产与社会化大市场的对接问题;实施财政金融扶贫，建立完善有效投融资机制，提高贫困地区和贫困人口的金融服务水平;实施基础设施建设扶贫，坚持科学规划，因地制宜解决通路、通水、通电、通网络等问题，使贫困地区基础设施得到明显提升;实施安居扶贫，把解决贫困人口脱贫与科学推进新农村建设结合起来，结合产业扶贫，坚持“五规合一”，优化村庄布局，合理规划建设公共服务设施，提升基本公共服务水平。

强化 “五个保障”。强化各级党委、政府的责任，层层压实责任，级级传导压力，真正把脱贫责任扛在肩上，把扶贫任务抓在手上;发挥基层党组织的战斗堡垒作用，选好村级组织带头人，增强引领群众脱贫致富能力;强化部门支持服务职能，帮助贫困地区想路子、抓产业、强保障，发挥好行业扶贫的作用;广泛动员社会力量扶贫，鼓励支持企事业单位、社会组织、个人参与脱贫攻坚，加快形成全社会参与的大扶贫格局;建立完善考评考核机制，通过考核考评，引导贫困地区党政领导班子和领导干部把工作重点和精力放在脱贫攻坚上，把工作做实、做准。

(八)切实保障和改善民生，促进社会和谐稳定。以实施重点民生工程为抓手，切实加大民生投入，强化薄弱环节建设支持力度，加快完善基本公共服务，维护社会和谐稳定。

促进就业和居民增收。继续实施好高校毕业生就业促进计划，强化困难人员就业援助，确保新增城镇就业5万人，新增农村劳动力转移就业6万人，失业人员实 现再就业1.99万人。多渠道增加农民经营性、工资性和财产性收入。持续增加城镇中低劳动者收入。落实好县以下机关建立公务员职务与职级并行制度和乡镇工 作补贴。

做好社会保障工作。深入实施社会保障体系完善工程，推动社会保障由制度全覆盖向法定人员全覆盖，确保五项社会保险参保率在90% 以上。继续深化机关事 业单位养老保险制度改革，切实做好城镇职工基础养老金全国统筹前期准备工作。统筹推进城乡社会救助体系建设，完善最低生活保障、特困人员供养、残疾人等兜 底制度，全面落实城乡低保发放、临时困难救助、农村“五保”供养等政策，健全关爱“三留守”人员工作机制。加快推进棚户区改造，开工建设棚户区改造项目 21个1.29万套。

提高教育发展水平。继续实施第二期学前教育三年行动计划，加快义务教育学校标准化建设，继续做好农村义务教育学校布局调整工作，深入推进农村义务教育学校“全面改薄”和扩充城镇义务教育资源五年规划，完成中心城区4所小学改扩建任务，开工建设1所中学和4所小学;积极推进普及高中阶段教育，落实好中专教育相关政策;加快推进职业技术学院新校区建设和现校区改高中项目，加快市职教园区农校、卫校、财校建设，确保如期搬迁入住;加大力度支持黄淮学院产教融合、转型发展和创新创业，承办好第三届产教融合发展战略国际论坛。

推进健康驻马店建设。深化医药卫生体制改革，加快县级公立医院综合改革步伐，巩固和完善基层医疗机构运行新机制，着力构建定位明确、分工协作的县域医 疗服务体系。完善城乡居民基本医保、城镇居民大病保险、新农合大病保险制度，全面实施疾病应急救助制度。加快市妇女儿童医院建设进度，市中医院儿科病房综 合楼完成主体工程，开工建设北区医院。坚持计划生育基本国策，落实全面两孩政策，开展优生优育全程服务，综合治理出生人口性别比失衡问题。加快养老服务体 系建设，促进医养结合，积极推进健康产业园区建设。

加快发展各项社会事业。深入实施农村电影放映工程”、“舞台艺术送农民”、“农家书屋”、“三馆一站免费开放”、“千场电影进社区进广场”等文化惠民 工程。健全公共文化服务体系，统筹用好工会服务职工等群众活动阵地和设施，坚持公益属性，真正发挥作用。加强基层文化服务中心建设，加快推进市气象科普 馆、科技馆、青少年宫等项目建设，加快西平、正阳、泌阳文化艺术中心和上蔡县博物馆筹建进度。加强公共体育设施建设，广泛开展全民健身活动，承办好河南省 “三山同登”全民健身大会，实施好农民体育健身工程、乡镇体育健身工程。加快养老服务体系建设，加强对社会办养老服务机构的管理和支持，积极推进社区和居 家养老服务设施建设，扎实做好农村敬老院社会化改革试点工作。加强残疾人就业培训，做好0~6岁残疾儿童抢救性康复工作，积极争创全国残疾人工作先进单 位。完善扶持政策，推动慈善事业健康发展。

维护社会安全稳定。着眼“双安”、推进“双治”、强化“双基”，进一步完善社会治理。加强社会治安立体防控体系建设，完善反恐怖工作和打击犯罪机制， 强化流动人口和特殊人群服务管理。认真落实属地管理和监管责任，着力解决重点区域、重点行业非法集资问题。继续做好信访工作，更加注重依法化解社会矛盾， 着力解决土地征用、房屋拆迁等民生领域的突出问题。严格落实安全生产责任制，深入开展重点行业、重点领域安全生产专项整治，切实加强食品药品监管，坚决遏 制安全生产和食品药品安全重特大事故发生。

(九)加强生态环境保护，打造美丽驻马店。坚持发展和保护两手抓，继续实施蓝天、碧水、乡村清洁工程和城市河流清洁行动计划，努力打造美丽驻马店。

加强环境保护和治理。实施蓝天工程，突出抓好燃煤和工业大气污染防治、机动车污染控制、扬尘综合治理、农作物秸秆禁烧及综合利用，淘汰和改造不符合要 求的燃煤锅炉，做好黄标车和老旧车淘汰工作。实施碧水工程，抓好重点流域水污染防治，全面实施城市河流清洁行动计划，开展城市河道综合整治，完成中心城区 练江河、开源河、朱氏河、黄酉河截污工程和各县城黑臭水河治理，加快推进市第二污水厂二期工程、再生水工程和第四污水处理厂项目，开工建设市生活垃圾无害 化综合处理再生利用、市建筑垃圾综合处理项目;加强饮用水源地保护，强化地表和地下水污染防治，保障饮用水安全;加强种植、畜禽养殖等农业面源和重金属污染防治，推进土壤污染治理。

促进资源节约和循环利用。实施重点领域节能减排降碳行动计划，支持企业实施节能环保技改及新上项目采用先进工艺技术，落实总量减排计划，强化重点减排 工程建设，加强减排项目运行监管。加快园区循环化改造和静脉产业园建设，推进建筑能效提升和绿色建筑全产业链发展计划，实施工业余热暖民和节能节水行动计 划。

抓好生态建设和保护。做好生态环境保护规划编制工作，统筹推进中心城区、西部山地生态区、东部平原涵养区、汝河涵养带、洪河涵养带和沿淮生态走廊规划 编制，努力构建集多层次、网络化、功能复合的生态系统。加强生态系统修复和建设，做好板桥水库、宿鸭湖水库等流域生态修复工作。严格落实水资源管理制度， 切实加强水功能区水质监测和入河湖排污口的监督管理。以国家森林城市创建为抓手，抓好天然林保护、退耕还林、城郊防护林等项目建设，完成造林100万亩。 持续加强生态保护，认真落实属地管理责任，健全党政领导干部生态环境损害责任追究制，落实排污单位主体责任和行业管理部门监管责任，实行一票否决并严肃追 究怠政失职。完善生态补偿机制，在环境高风险领域建立污染强制责任保险制度，引入第三方治理。完善生态环境突发性灾害事件应急机制。

加强国防教育和国防后备力量建设，支持国防和军队改革，支持驻驻解放军、武警部队、预备役部队、民兵和人民防空建设，深入开展双拥共建，推进军民融合 发展。支持工会、共青团、妇联等人民团体发挥桥梁纽带作用，注重发挥工商联等社团作用。认真做好民族宗教、外事侨务、统计、档案、史志、气象、地震、对口 援疆等工作。

根据《驻马店市国民经济和社会发展第十三个五年规划纲要》，全面完成各专项规划编制工作。四、切实加强政府自身建设

认真贯彻法治政府建设实施纲要，把转变政府职能、强化依法行政、加强廉政建设、转变工作作风作为政府自身建设的关键，努力建设法治政府、创新政府、廉洁政府、服务政府、高效政府，全面提升政府治理能力和水平。

(一) 加快转变政府职能。正确处理政府和市场的关系，围绕市场在资源配置中起决定性作用和更好发挥政府作用，推动政府职能向创造良好发展环境、提供优 质公共服务、维护社会公平正义转变。进一步简政放权，严格执行行政审批目录清单，继续精简行政审批事项，清理规范行政审批中介服务，全面建立权责清单并实 行动态管理。加强和改善事中事后监管，推开“两随机、一公开”检查，切实把该管的管好。深化政务公开，依法及时准确地向社会公开政府信息，确保权力公开透 明、规范运行。

(二)大力推进依法行政。严格按照法定权限和程序行使权力、履行职责，切实做到法定职责必须为，法无授权不可为。坚持和完善公众参与、专家论证、风险 评估、合法性审查和集体决策相结合的决策程序。积极推进行政综合执法体制改革，推行服务型行政执法。启动“七五”普法。自觉接受人大及其常委会法律和工作 监督，主动接受政协民主监督，严格执行人大及其常委会决定，认真办理人大代表建议和政协提案，拓宽参政议政渠道，广泛听取民主党派、工商联、无党派人士、 人民团体意见，自觉接受社会监督和舆论监督，让人民群众更有效地监督政府，让行政权力始终在法治轨道和阳光下运行。

(三)切实加强廉政建设。坚持纪严于法、纪在法前，严明政治纪律和政治规矩，履行主体责任和“一岗双责”，落实党风廉政建设责任制，把廉洁从政贯彻到 政府工作各个环节。强化权力运行制约监督，加强行政监察和审计监督，强化巡视巡察监督，进一步完善财政资金支出、工程项目招投标、土地出让、行政审批、公 共资源交易等领域的监督制度，坚决打掉寻租空间，努力铲除腐败土壤。强化廉政教育，严格执行廉政准则和各项规定，始终保持清廉本色，以反腐倡廉实效保障发 展、取信于民!

(四)持续转变工作作风。践行“三严三实”要求，认真落实中央八项规定和省市实施意见，开展懒政怠政为官不为问责年活动，从市政府领导班子做起，以上率下，全面加强政府作风建设，坚持不懈纠正“四风”。 坚 持密切联系群众，问政于民、问需于民、问计于民，办好顺民意、解民忧、惠民生的实事。坚持一切从实际出发，脚踏实地，务实重干，不急功近利，不盲目跟风， 努力做出经得起实践、人民、历史检验的业绩。坚持简约从政，杜绝铺张浪费，压缩一切不必要开支，集中财力办大事，确保三公经费只减不增，确保把有限的财力 用到最急需的地方。

(五)着力抓好工作落实。不断加强公务员特别是各级领导干部的教育培训和实践锻炼，克服本领恐慌，进一步提高研判经济形势、应对复杂局面、化解疑难问 题的能力。健全岗位履职责任制和激励机制，督促引导各级公务人员履职尽责、勤政有为、真抓实干。大力实施模拟审批、联审联批、全程代理和网上审批办理，切 实提高办事效率和服务水平。牢固树立法治思维法治方式、市场思维市场方式，对影响工作推进的关键问题、制约长远发展的重大难题、群众反映强烈的热点问题， 坚持用改革创新的办法进行破解。特别是对于今年确定的各项重点项目、重点工作，建立工作台账，加大督促检查，强化行政问责，切实提高抓落实的实效性。下决 心打通政策落实中的“中梗阻”和“最先最后一公里”，真正形成倒逼机制，确保各项工作落到实处。

各位代表!

全面完成 2016年目标任务，责任重大，使命光荣。让我们紧密团结在以习近平同志为总书记的党中央周围，在省委、省政府和市委的坚强领导下，进一步解 放思想、团结奋进、锐意进取，奋力开创全市经济社会跨越发展新局面，为实现“十三五”开门红，建设富强、文明、平安、美丽驻马店而努力奋斗!
